# Comorbidities and mortality risk in adults younger than 50 years of age with chronic obstructive pulmonary disease

**DOI:** 10.1186/s12931-022-02191-7

**Published:** 2022-09-27

**Authors:** Miguel J. Divo, José M. Marin, Ciro Casanova, Carlos Cabrera Lopez, Victor M. Pinto-Plata, Marta Marin-Oto, Francesca Polverino, Juan P. de-Torres, Dean Billheimer, Bartolome R. Celli, The BODE Collaborative Group, Bartolome R. Celli, José M. Marin, Ciro Casanova Macario, Victor Pinto-Plata, Juan Pablo de-Torres, Miguel J. Divo, Carlos Cabrera Lopez, Francesca Polverino, Marta Marin Oto

**Affiliations:** 1 Pulmonary and Critical Care Division, Brigham and Women’s Hospital and Spaulding Rehabilitation Hospital, Harvard Medical School, 75 Francis Street, Boston, MA 02115 USA; 2 Respiratory Service, Hospital Universitario Miguel Servet-IISAragón & CIBER Enfermedades Respiratorias, Avda Isabel la Catolica 1-3, 50006 Saragossa, Spain; 3 Pulmonary Department, Hospital Universitario La Candelaria, Universidad de La Laguna, Carretera del Rosario n 145, 38010 Santa Cruz de Tenerife, Spain; 4 Respiratory Service, Hospital Universitario de Gran Canaria Dr. Negrin, Las Palmas, Canary Islands Spain; 5 Pulmonary and Critical Care Division Chair, Lahey Hospital and Medical Center, Burlington, MA USA; 6 Pulmonary Department, Hospital Clínico Universitario Lozano Blesa, Saragossa, Spain; 7 Department of Medicine, Baylor College of Medicine, Houston, TX USA; 8 Division of Respirology and Sleep Medicine, Queen’s University, Kingston, Canada; 9 BIO5 Institute, Mel and Enid Zuckerman College of Public Health, University of Arizona, Tucson, AZ USA

**Keywords:** COPD in the young, Comorbidities

## Abstract

**Rationale and objective:**

Patients with chronic obstructive pulmonary disease (COPD), usually diagnosed after the 6th decade, frequently suffer from comorbidities. Whether COPD patients 50 years or younger (Young COPD) have similar comorbidities with the same frequency and mortality impact as aged-matched controls or older COPD patients is unknown.

**Methods:**

We compared comorbidity number, prevalence and type in 3 groups of individuals with ≥ 10 pack-years of smoking: A Young (**≤ **50 years) COPD group (n = 160), an age-balanced control group without airflow obstruction (n = 125), and Old (> 50 years) COPD group (n = 1860). We also compared survival between the young COPD and control subjects. Using Cox proportional model, we determined the comorbidities associated with mortality risk and generated Comorbidomes for the “Young” and “Old” COPD groups.

**Results:**

The severity distribution by GOLD spirometric stages and BODE quartiles were similar between Young and Old COPD groups. After adjusting for age, sex, and pack-years, the prevalence of subjects with at least one comorbidity was 31% for controls, 77% for the Young, and 86% for older COPD patients. Compared to controls, “Young” COPDs’ had a nine-fold increased mortality risk (p < 0.0001). “Comorbidomes” differed between Young and Old COPD groups, with tuberculosis, substance use, and bipolar disorders being distinct comorbidities associated with increased mortality risk in the Young COPD group.

**Conclusions:**

Young COPD patients carry a higher comorbidity prevalence and mortality risk compared to non-obstructed control subjects. Young COPD differed from older COPD patients by the behavioral-related comorbidities that increase their risk of premature death.

**Supplementary Information:**

The online version contains supplementary material available at 10.1186/s12931-022-02191-7.

## Background

Chronic obstructive pulmonary disease (COPD) is usually diagnosed in individuals older than 60 years. However, some individuals are diagnosed at age 50 or younger [1–3]. This group of younger COPD patients has been named “Early-onset COPD” [1], “Young COPD” [2], and more recently, Martinez et al. [4] proposed the term “Early” COPD. By consensus, they defined this group as subjects 50 years of age and younger with at least 10 pack-years of cumulative smoking and airflow limitation, lung structural alterations by computed tomography, or accelerated lung function decline [4]. This subgroup of COPD patients has a similar degree of airflow limitation, rate of lung function decline, and BODE scores compared to those older than 65 years of age [2].

COPD rarely presents as the only disease affecting a patient, as more than 80% of COPD patients have one or more comorbidity [5]. Besides, young subjects with poor lung function have a higher risk of developing some diseases such as cardiovascular comorbidities and diabetes approximately 10 years earlier than individuals with normal lung function [6]. Using a hypothesis-free system network analysis in almost 2000 COPD patients, we found that their recorded comorbidities tended to cluster differently around specific clinical characteristics. In particular, we observed a cluster of psychiatric and behavioral comorbidities in the subset of COPD patients younger than 55 years of age [7], an observation that was similar to that reported by Vanfleteren and co-workers in 213 COPD patients participating in a rehabilitation program [8]. These investigators described one of five clusters, characterized by a higher prevalence of anxiety and depression [8]. Taken together, this suggests that COPD patients younger than 50 years may have similar pulmonary dysfunction as their older counterparts, but the prevalence, pattern, and impact of comorbidities might be different.

We conducted this study using patients enrolled in the BODE cohort prospective registry to test two hypotheses. First, that COPD patients younger than 50 years (Young COPD) carry a higher burden of comorbidities and mortality risk than ever-smoker controls of similar age who do not have chronic airflow obstruction. Second, that comorbidities associated with a higher mortality risk differ in Young COPD patients compared to those COPD individuals older than 50 years.

## Methods

### Participants

The BODE cohort is an ongoing observational prospective registry, following individuals at risk or with established COPD attending pulmonary clinics at BODE study centers (Tampa and Boston in the USA, Pamplona, Tenerife, Las Palmas de Gran Canaria, and Zaragoza in Spain) [9]. For this study, we included COPD subjects enrolled between January 1997 and December 2015, and volunteers without obstruction (“controls”) recruited in the same centers. All participants had at least 10 packs/years of cumulative smoking, and for subjects with COPD, they all met the ATS/ERS standards for the diagnosis of the disease [10]. All spirometries were performed following international standards [11]. Like other COPD cohorts, from 1997 to 2010, the BODE cohort excluded individuals diagnosed with a concomitant diagnosis of asthma. For the current analysis, individuals with alpha-1 antitrypsin (AAT) deficiency determined by serum levels of ATT < 20 μmol/L or < 100 mg/dL were also excluded. We recorded the baseline anthropometric, smoking history, spirometry, and BODE index scores [9]. All participants were in clinically stable conditions, and for COPD subjects, receiving standard therapy according to contemporary guidelines [12–15]. The ethics committee at each participating center approved the study, and all subjects signed informed consent.

### Definitions

Based on the post-bronchodilator spirometry, subjects were designated as having normal spirometry if the FEV_1_/FVC ≥ 0.70 and FEV_1_ percent predicted ≥ 80%, and COPD when the FEV_1_/FVC is < 0.70 and the severity of obstruction based on the FEV_1_ percent predicted value according to the Global Initiative for Chronic Obstructive Lung Disease (GOLD) classification [15]. For this study, we used the term “Young COPD” for those subjects diagnosed with COPD at age 50 or younger with 10 or more pack-years of smoking history, as consensually agreed by Martinez and co-workers [4]. All subjects with COPD older than 50 years of age and the same smoking criteria were defined as “Old” COPD. Subjects with 10 or more pack-years of smoking who are 50 years or younger without chronic airflow limitation were defined as “control” subjects. We adhered to the GOLD definition of chronic airway obstruction (FEV_1_/FVC < 0.70) as its provide accurate discrimination for important COPD related outcomes [16].

### Comorbidities

During the initial visit interview, comorbidities were systematically recorded through participant’s interviews or as documented in their medical records. The presence of each comorbidity was then confirmed at each site through a detailed review of confirmatory tests when applicable (for example ECG, echocardiogram, etc.), medications use, or therapy specific to each specific diagnosis [7, 17]. For this analysis, comorbidities were tabulated as the presence or absence of the disease.

### Follow-up and cause of death

After enrolment, participants were followed prospectively for at least 5 years or until death. We verified death or loss to follow-up by calling each subject or their family if they failed to return for appointments. We investigated the principal cause of death as previously published [9]. Each enrolment site ascertained the cause-specific mortality to the highest possible detail and then categorized it as either related to (1) COPD, (2) non-COPD respiratory cause, (3) cardiovascular, (4) cancer, (5) other cause, or (6) unknown.

### Statistical analysis

Data are summarized as mean and standard deviation (SD) or median and interquartile range (IQR) for continuous variables according to their distribution. Categorical or binomial variables are presented as proportions. We used two-sample t-tests and Fisher’s exact test to compare two groups, depending on the variable type.

#### Comorbidities burden

We estimated the proportion of individuals with 0, 1, 2, 3, and 4 or more comorbidities based on number of comorbidities at time of recruitment. Using an ordinal logistic regression model, we tested the hypothesis that the proportion of subjects with 0, 1, 2, 3, or 4 or more comorbidities were different for each of the three groups (Young COPD, control, or Old COPD). The model was adjusted for the following confounders, age, gender, current smoking status, and cumulative smoking (pack-years) to account for the differences in their baseline characteristics.

#### Prevalence comparison

We tested if comorbidities prevalence differed between Young COPD and control subjects using logistic regression adjusting by age, gender, smoking status, and pack-years of smoking. We separately compared comorbidities prevalence between the Young and Old COPD group, adjusting for gender, smoking status, cumulative smoking. Comorbidities’ prevalence was calculated based on those comorbidities present during the initial visit.

#### Survival analysis and the “Comorbidomes”

To evaluate the strength of the association between the comorbidities and all-cause mortality, we performed multivariate analyses using Cox proportional hazard regression for the Young COPD and separately for the Old COPD group. From the 83 different comorbidities recorded, we reduced the number of candidate comorbidities for the Cox model by excluding those with less than 2% prevalence and by selecting the most plausible comorbidities based on clinical knowledge and applying bootstrap forest partitioning as a variable reduction method. Once comorbidities were identified, we used backward elimination keeping those with significant association with mortality. Hazard ratios (HR) with 95% confidence intervals (CI) were estimated. Time of follow-up was used as time scale. We integrated this information with disease’s prevalence to construct the “Comorbidomes” as previously published [17]. The Comorbidome is an orbital representation of the prevalence of comorbidities and their associated risk of death. We estimated the survival probability at 3, 5, and 10-years between the three groups using a Kaplan–Meier analysis, and the statistical significance determined by the log-rank test.

For hypothesis testing, a p < 0.05 was deemed statistically significant. We used SAS JMP Pro^®^ software, version 14.3 (SAS Institute), for all analyses.

#### Sensitivity analysis

Since we included for this study individuals recruited from 1997 to the year 2015, and from two different countries, we conducted a sensitivity analysis accounting for those factors. We divided the recruitment time span in three epochs, as those recruited from 1997–2000 (n = 773), 2001–2005 (n = 794), and 2006–2015 (n = 578); for recruitment site, as well as those recruited in Spain or in the US.

## Results

Of the 2145 enrolled participants, 160 subjects met the criteria for Young COPD, 125 were control subjects, and 1,860 Old COPD. Table 1A and B show the characteristics of the cohort at enrolment. The control group was, on average, slightly younger than the Young COPD patients (43 ± 5 years vs. 46 ± 4 (p = *0.0001*), with an age range from 34 to 50, and 29 to 50 years, respectively. The proportion of females also differed between groups, and there were more current smokers in the control group but with a lower cumulative amount of smoking (pack-years). Older COPD subjects had in average lower FEV_1_% predicted (51 ± 21 vs 61 ± 27, *p* < *0.0001*), and higher BMI than Young COPD patients. However, the proportion of subjects distributed among the GOLD categories, mean BODE score, and quartiles were similar between these two groups (Table 1B).

**Table 1:** Subjects’ baseline characteristics

A			
Demographic	Control subjects	Young COPD ≤ 50 years	*p*-value
N	125	160	
Age (mean, SD)	43 ± 5	46 ± 4	*0.0001*
Male (%)	52%	76%	< *0.0001*
Current smoker (%)	76%	61%	*0.001*
Pack-years (mean, SD)	28 ± 14	49 ± 31	*0.0001*
FEV_1_/FVC (mean, SD)	0.81 ± 0.05	0.57 ± 0.15	< *0.0001*
FEV_1_% predicted (mean, SD)	101 ± 2	61 ± 2	< *0.0001*
BMI (Kg/m^2^) (mean, SD)	26.4 ± 4.9	26.2 ± 5.8	*0.9181*

B			
Demographic	Young COPD ≤ 50 years	Old COPD > 50 y/o	*p*-value
N	160	1860	
Age (mean, SD)	46 ± 4	68 ± 8	< *0.0001*
Male (%)	76%	90%	< *0.0001*
Current smoker (%)	61%	32%	< *0.0001*
Pack-years	49 ± 31	76 ± 45	< *0.0001*
FEV_1_/FVC (mean, SD)	0.57 ± 0.15	0.49 ± 0.14	< *0.0001*
FEV_1_% predicted (mean, SD)	61 ± 27	51 ± 21	< *0.0001*
GOLD categories (all with FEV_1_/FVC < 0.70)			
GOLD I (FEV_1_ ≥ 80%)	15%	10%	0.2842
GOLD II (FEV_1_ 50–79%)	33%	39%	
GOLD III (FEV_1_ 30–49%)	33%	36%	
GOLD IV (FEV_1_ < 30%)	19%	15%	
BMI (kg/m^2^) (mean, SD)	26.2 ± 5.8	27.3 ± 5.4	*0.0289*
BODE score (mean, SD)	2.9 ± 2.4	3.4 ± 2.6	0.1012
BODE score 0 to 2	48%	42%	*0.3393*
BODE score 3 and 4	29%	26%	
BODE score 5 and 6	13%	17%	
BODE score 7 to 10	10%	15%	

(A) Compares the baseline characteristics of Young COPD participants and current and ex-smokers without baseline chronic airway obstruction (Controls). (B) Compares the baseline characteristics of Young COPD (≤ 50 years) and Old COPD subjects (51-years and older)

*6MWT* six-min walk test, *BMI* Body Mass Index, *BODE* BMI-Obstruction-Dyspnea-Exercise index, *BODE* Body mass index, Obstruction, Dyspnea and Exercise capacity index, *FEV*_*1*_ Forced Expiratory volume in 1st second, *FVC* forced vital capacity, *GOLD* Global Initiative for Chronic Obstructive Lung Disease

### Comorbidities burden

The ordinal logistic regression model demonstrated that having COPD is the strongest predictor for a higher number of comorbidities, with Old COPD carrying the most comorbidities, followed by the Young COPD group, while the control group had the lowest number of comorbidities (p < 0.0001). The other factors associated with a higher number of comorbidities within each group were the cumulative amount of smoking, male gender, and older age (p < 0.0001).

**Fig. 1:**
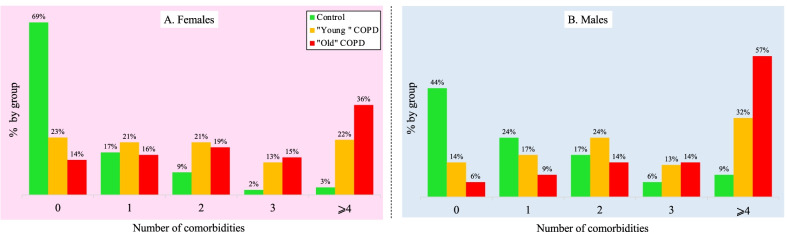
The proportion of individuals carrying 0, 1, 2, 3, or ≥ 4 concurrent comorbidities within each group. The Right panel (pink) represent the estimates for females and the left panel (blue) for males. We adjusted the proportions estimates for age and cumulative smoking (see text for details)

Figure 1 shows the proportion of individuals in each of the three groups carrying 0, 1, 2, 3, and 4 or more comorbidities; these estimates were *adjusted* for females (left panel in pink) and males (right panels in blue), estimated for an individual aged 45 years and a 35 pack-year of smoking. The same figure includes the cumulative proportion of comorbidities in the older COPD group (male, with an average age of 60 years and 35 pack-years of smoking). In females, we estimated that 69% of control subjects had *no* chronic disease at baseline, while only 23% of Young COPD and 14% of Old COPD had no comorbidity other than COPD. In contrast, 22% of the Young COPD and 36% of Old COPD females had four or more comorbidities compared to only 3% in the female controls (Fig. 1 left panel). A similar finding was obtained for males in the three groups (Fig. 1 right panel).

**Fig. 2:**
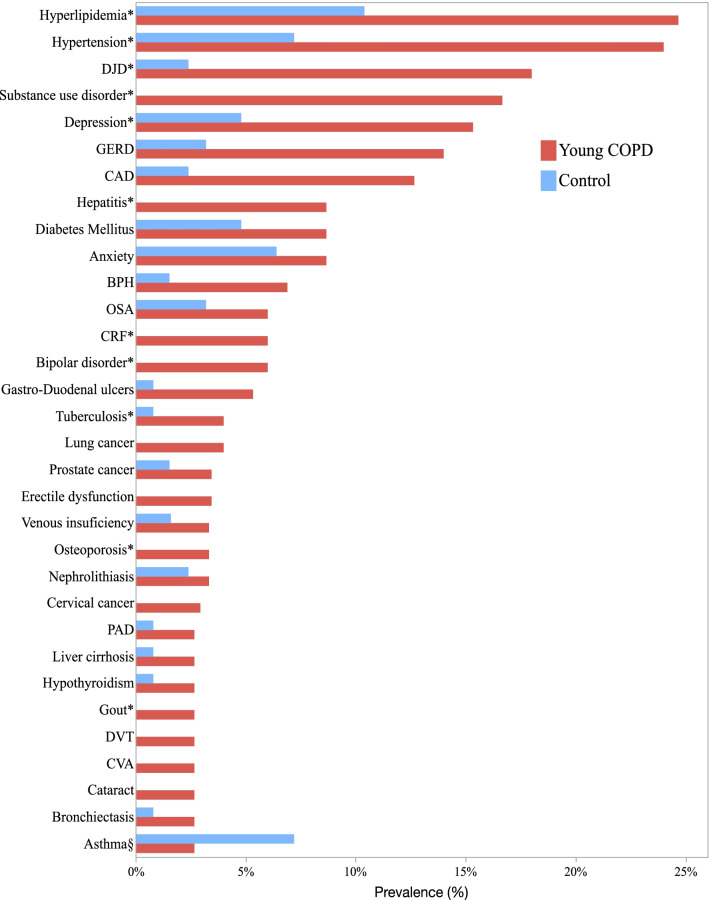
Comparison of comorbidities prevalence between Young COPD and control subjects. The figure shows only those comorbidities with at least 2% prevalence. An asterisk (*) denotes that the difference between the disease’s prevalence in the two groups is statistically significant after adjusting for age, gender, and cumulative smoking. § In the COPD groups, asthma diagnosis was accounted for those recruited after 2010, as it was an exclusion criterion in the initial BODE cohort protocol. Substance use disorder comprise alcohol, hallucinogens, stimulants, sedatives and/or opioids use. *AAA* Abdominal aortic aneurism, *BPH* Benign prostatic hypertrophy, *CA* cancer, *CAD* Coronary artery disease, *CHF* congestive heart failure, *CRF* chronic renal failure, *CTD* connective tissue disorders, *CVA* cerebro-vascular accident, *DJD* Degenerative joint disease, *DVT* deep venous thrombosis, *GERD* gastro-esophageal reflux disease, *OSA* obstructive sleep apnea, *PAD* peripheral artery disease, *PH* pulmonary hypertension

Comorbidities prevalence between Young COPD and control subjects are shown in Fig. 2 and Additional file 1: Table S1, and between Young and Old COPD subjects in Additional file 1: Fig. S1 and Table S2. The Young COPD group have a higher crude prevalence than control subjects except for asthma and connective tissue disorders (CTD). After adjusting for age, gender, smoking status, and the cumulative amount of smoking, the prevalence was statistically different in ten of those comorbidities, namely, hyperlipidemia, hypertension, degenerative joint disease, substance use disorder (as based on the definition by the Diagnostic and Statistical Manual of Mental Disorders (DSM-5), substance use disorder comprises alcohol, hallucinogens, stimulants, sedatives and/or opioids use), depression, hepatitis, bipolar disorder, chronic renal failure, tuberculosis, and osteoporosis, all marked with an asterisk in Fig. 2 and Additional file 1: Table S1 [18].

When comparing the two COPD groups, the crude prevalence of most comorbidities was higher in the Old COPD group (Additional file 1: Fig. S1 and Table S2). However, after adjusting for the differences in gender, smoking status, and cumulative smoking, the difference was statistically higher in 18 out of the 83 comorbidities for the older group (marked with an asterisk in Additional file 1: Fig. S1). Conversely, the prevalence of hepatitis was statistically higher for the Young COPD group.

### Survival analysis

Vital status was confirmed in 1828 of the 2145 participants, with data missing in 7 “Young COPD”, 16 controls, and 294 older COPD. Over the median observation time of 10 years (IQR 5.4–15.8) we recorded 690 total deaths, of which 6 were among the controls, 35 for the “Young COPD”, and 649 among the older COPD. The Kaplan–Meier survival curves are presented in Additional file 1: Fig. S2, and the crude survival estimates (95% CI) at 3, 5, and 10 years were 100% for the control group (recorded deaths occured after 16 years of follow-up), while for the “Young COPD” they were 92% (86–95%) at 3, 83% (76–89%) at 5, and 69% (58–77%) at 10 years. For the older COPD group, the estimates were 77% (75–79%), 62% (59–64%), and 38% (33–43%) respectively and the differences between groups are significant (p < 0.0001). For the adjusted survival analysis (Cox’s proportional hazard), we compared survival between the Young COPD with control subjects; Old COPDs were excluded because of age’s strong confounding effect as a predictor of death. After adjusting for gender, age, and cumulative smoking, the strongest predictors for mortality were, belonging to the Young COPD group (hazard ratio of 9.01 [95% CI 6.10–13.3], p < 0.0001), and by the cumulative smoking (pack-years) (hazard ratio of 1.007 for every 1 unit increase in pack-year [95% CI 1.001–1.012], p = 0.0144).

The leading causes of death in both the Young COPD and Old COPD group were cancer and COPD (Additional file 1: Fig. S3).

**Fig. 3:**
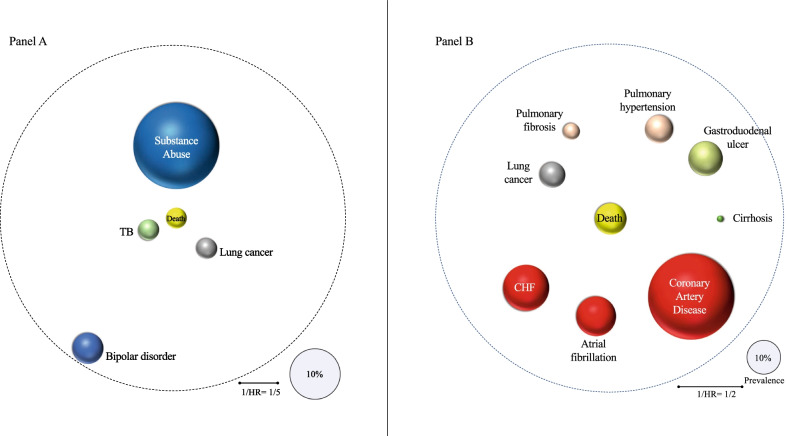
Representation of the “Comorbidomes” for both the Young COPD patients ≤ 50 years (**A**) and Old COPD > 50 years of age (**B**). The bubbles’ sizes are proportional to the comorbidity prevalence, and the distance to the center is proportional to the inverse of the hazard ratio (1/HR) for death; the closer to the center, the higher is the association with death. See Table 2 for actual hazard ratio values. The comorbidities represented are those with significant association to death, and the dotted circular line represents the cut-off of statistical significance set at p < 0.05 for the hazard ratio

The comorbidities that demonstrated a statistically significant association with mortality in the Young and Old COPD patients are presented as “Comorbidomes” in Fig. 3 and the estimated hazard ratios in Table 2. In both groups, lung cancer was significantly associated with mortality. In contrast, history of tuberculosis, bipolar disorder, and substance use disorder were specific for the Young COPD patients, while pulmonary fibrosis, liver cirrhosis, atrial fibrillation, coronary artery disease, congestive heart failure, pulmonary hypertension, and gastro-duodenal ulcers for the older group.

**Table 2:** Disease prevalence and its association with mortality risk in Young and Old COPD patients

Comorbidity	Prevalence (%)	Hazard Ratio	Lower 95%	Upper 95%	p-value
1. Young COPD					
History of TB	4.0	8.35	2.02	34.47	0.0033
Bipolar disorder	6.0	7.71	1.86	31.97	0.0049
Lung cancer	4.0	5.78	1.15	28.83	0.0323
Substance use disorder	16.7	3.39	1.35	8.51	0.0093
2. Old COPD					
Lung cancer	8.1	2.16	1.74	2.69	< 0.0001
Pulmonary fibrosis	5.3	1.62	1.23	2.13	0.0006
Liver cirrhosis	2.1	1.59	1.03	2.48	0.0360
Atrial fibrillation	12.6	1.44	1.16	1.77	0.0007
CAD	27.3	1.41	1.19	1.68	< 0.0001
CHF	14.6	1.34	1.09	1.67	0.0058
Pulmonary hypertension	8.9	1.27	1.15	1.80	0.0311
Gastroduodenal ulcers	10.9	1.23	1.01	1.54	0.0454

*CAD* coronary artery disease, *CHF* congestive heart failure, *TB* tuberculosis

Sensitivity analyses using the year when subjects were recruited and stratified as 1997–2000 (n = 773), 2001–2005 (n = 794), and 2006–2015 (n = 578) did not significantly change the results (data not shown). Similarly, when adjusting by recruitment center country (US and Spain) the results did not differ.

## Discussion

To our knowledge, this is the first study to describe the prevalence and impact of comorbidities in individuals 50 years or younger with a confirmed diagnosis of COPD. Compared to ever-smoker subjects of similar age and without chronic airflow limitation, Young COPD subjects had a higher number and prevalence of comorbidities and a nine-fold higher mortality risk over the 10 years of observation. Compared with the Old COPD patients, the Young COPD patients with the same distributions of GOLD spirometric stages and BODE scores than Older patients had behavioral-related comorbidities (substance use and bipolar disorders) that specifically impacted on mortality in this Young group of patients.

### Co morbidities in Young COPD and controls

In this study, we observed that comorbidities are more frequent in Young COPD patients compared with ever smokers of similar age but without airflow obstruction. Indeed, 77% of the females and 86% of males had at least one chronic condition (excluding COPD) compared to 31% and 56%, respectively, in the control subjects. Also, we found a significantly higher prevalence in ten comorbidities in the younger COPD patients compared with control subjects (Fig. 2 and Additional file 1: Table S1). Among them, five comorbidities (degenerative joint disease, osteoporosis, systemic hypertension, chronic renal failure, and hyperlipidemia) are characteristically seen in the elderly [21]. Finally, after adjusting for covariates, young patients with COPD had a nine-fold increase in the risk of death compared with the non-obstructed controls.

For decades COPD has been thought to be a disease of the elderly. However, observations from different studies have challenged this concept. Morice et al. [19] identified 365 patients younger than 50 years, amongst the close to 6000 patients enrolled in the 4-year UPLIFT study (only patients older than 40 years were allowed into the study). The authors noted that the distribution of airflow limitation using the GOLD spirometric staging was similar between younger and older patients. Sanchez-Salcedo et al. [2] examined data from the BODE cohort and identified 103 COPD patients younger than 55 years and 463 older than 65 years and compared them at baseline and after 4 years of follow-up. In this study, the disease’s severity distribution and progression (lung function and BODE index change) in the younger COPDs’ were similar to those of older patients. Çolak et al. [3] found in the Copenhagen General Population Study an estimated 15% prevalence of airway obstruction among individuals younger than 50 years. More recently, Cosío et al. [20, 21] found that this group is underdiagnosed, despite having significant degree of emphysema, air trapping, and symptoms. Both independent studies by Çolak and Cosío demonstrated that this younger cohort had a worse health status, increased exacerbation risk, and poor outcomes [3, 20]. Nonetheless, no detail was made on the prevalence, type, and impact of comorbidities. In a large longitudinal population study, Jensen and colleagues [21] among other diseases identified COPD as an illness that preceded the occurrence of subsequent morbidities. Interestingly, in this study, mental and behavioral disorders anteceded COPD development.

### Comorbidities in the Young and Older COPD patients

As expected from the effect of age and the cumulative amount of smoking, Old COPD patients had more comorbidities and prevalence than the Young COPD group. However, we found that the comorbidities and their relationship to risk of death differed between groups. Indeed, the “Comorbidomes” between the “Young” and “Old” COPD groups were very different. While in both groups, lung cancer had a significant association with mortality, in the “Young” COPD group, history of tuberculosis, substance use disorders, and bipolar disorder were distinct comorbidities associated with higher mortality not seen in the older group. These differences suggest that while most of the diseases on the “Old” COPD comorbidome have a direct link with mortality, in the “Young,” we observe two discrete patterns: one characterized by tobacco-related disease (lung cancer) and a second characterized by behavioral and socio-economic-related diseases (substance use disorder, bipolar disorder, and history of tuberculosis). These findings highlight the importance of nature and nurture in the construct of COPD. Indeed, studies have demonstrated that poor lung function in early adulthood can aggregate in families and is associated with maternal smoking and lower birth weight [20]; if the vulnerable individual engages in smoking and other substance use disorder, the vulnerability to incident COPD is enhanced [1, 6, 22–24]. The impact of behavioral dysfunction on outcomes is not a phenomenon exclusive to COPD. Fatal drug overdoses, alcoholic liver disease, and suicide were linked to the recent decline in U.S. life expectancy, mainly affecting adults in their midlife [25]. Multiple US population surveys have found that about half of those who experience a mental illness during their lives will also experience a substance use disorder and vice versa [26]. Further, it is known that nicotine, alcohol, and other illicit substance related disorders have a higher co-occurrence with major depression and other psychiatric illnesses [22]. In support of this association, our study found the prevalence of major depression to be 15% in both COPD groups while only 5% in non-COPD controls. The findings in our study suggests that behavioral problems and particularly substance abuse disorder should be considered in any patient diagnosed with COPD at a younger age.

## Strengths and limitations

This study has several strengths, including the relatively large number of “Young” COPD, the study’s multicenter design, the prolonged observation period, and the spectrum of different comorbid conditions assessed. However, it also had limitations. First, the BODE cohort recruits mainly subjects attending pulmonary clinics; therefore, the results represent a segment of the smoking-related COPD population and some smokers without obstruction. Hence, our results require replication in population-based cohorts. Second, since its inception until 2010, the BODE cohort has excluded patients with asthma defined as a bronchodilator response in FEV_1_ of more than 15% or 200 ml and 12% above baseline [9]. This condition is likely to explain the relatively low prevalence of asthma in the Young COPD compared to other reports [3, 27] as well as in the controls in this study. Third, the control group differed statistically in age, gender composition, and cumulative cigarette smoking compared to the Young COPD group. However, we corrected our estimates by adjusting for those confounders in all regression models. Fourth, our estimates are from comorbidities tabulated on the initial study visit (prevalence); therefore, we cannot establish incidence trajectories. Sixth, there is a considerable time span among participants recruited the BODE cohort, as some individuals were recruited in the late 1990’s up to 2015. To overcome potential bias, we conducted sensitivity analysis, and we did not find differences in our conclusion. Finally, we do not have reliable information about early life events or diseases that influence lung growth, or occupational exposures that can affect COPD risk and associated comorbidities [28].

In conclusion, Young COPD patients carry a high burden of comorbidities some of which are specifically frequent in this diagnostic group. These morbidities confer an increased risk of death at this early age. Our findings support the need to identify airway obstruction at a younger age and identify and address those behavioral comorbidities that confer increased mortality risk in a young patient with COPD.

## Supplementary Information

**Additional file 1: Figure S1.** Comorbidities prevalence bar graph comparing Young (≤ 50 years) and older (> 50 years) COPD patients. **Figure S2.** Kaplan–Meier survival curve comparing the three groups. In blue is the control group, red for “Young COPD” and green for the older COPD. **Figure S3.** Primary causes of death in the Young (*<* 50 years) and “Older” COPD patients (> 50 years). **Table S1.** Comorbidities prevalence comparison between Young COPD and controls. **Table S2.** Comorbidities prevalence comparing Young COPD and Old COPD.

## Data Availability

The datasets generated and/or analyzed during the current study are not publicly available but could be available from the corresponding author on reasonable request.

## Abbreviations

BODE: The Body Mass Index, Obstruction, Dyspnea, and Exercise Index
CI: Confidence interval
COPD: Chronic obstructive pulmonary disease
CTD: Connective tissue disorders
FEV_1_: Forced expiratory volume in the first second
HIV: Human immunodeficiency virus
IQR: Interquartile range
