# Supplementary material for: Comorbidities and mortality risk in adults younger than 50 years of age with chronic obstructive pulmonary disease
Source: Respir Res. 2022 Sep 27;23:267. doi: 10.1186/s12931-022-02191-7 (PMC9516817; doi:10.1186/s12931-022-02191-7)
Supplement: Supplementary file 1 — Additional file 1: Figure S1. Comorbidities prevalence bar graph comparing Young (≤ 50 years) and older (> 50 years) COPD patients. Figure S2. Kaplan–Meier survival curve comparing the three groups. In blue is the control group, red for “Young COPD” and green for the older COPD. Figure S3. Primary causes of death in the Young (< 50 years) and “Older” COPD patients (> 50 years). Table S1. Comorbidities prevalence comparison between Young COPD and controls. Table S2. Comorbidities prevalence comparing Young COPD and Old COPD. [file 12931_2022_2191_MOESM1_ESM.docx]

**Supplemental Material**

Comorbidities and Mortality Risk in Adults Younger than 50 years of age with Chronic Obstructive Pulmonary Disease

**Authors:**

Miguel J. Divo, MD MPH, José M. Marin, MD, Ciro Casanova Macario, MD, Carlos Cabrera Lopez, MD, Victor M. Pinto-Plata, MD, Marta Marin-Oto, MD, Francesca Polverino, MD PhD, Juan P. de-Torres, MD, Dean Billheimer, PhD, and Bartolome R. Celli, MD.

The BODE Collaborative group

Content:

Figures:

1. e-Figure 1. Comorbidities prevalence bar graph comparing Young (≤ 50 years) and Old COPD (> 50 years) patients.
2. e-Figure 2. Primary causes of death in the Young and Old COPD patients.

Tables:

1. e-Table 1. Comorbidities prevalence comparison between Young COPD and controls.
2. e-Table 2. Comorbidities prevalence comparing Young COPD and Old COPD.

e-Figure 1. Comorbidities prevalence bar graph comparing Young ( <50 years) and older (>50 years) COPD patients.

*The bar graph includes those comorbidities with at least 2% prevalence; for a full list refer to e-Table 2.*

*Footnote: Abbreviations: AAA= Abdominal Aortic Aneurism, BPH= Benign Prostatic Hypertrophy, CA= Cancer, CAD = Coronary Artery Disease, CHF=Congestive Heart Failure, CRF=Chronic Renal Failure, CTD= Connective Tissue Disorders, CVA=Cerebro-Vascular Accident, DJD= Degenerative joint Disease, DVT= Deep Venous Thrombosis, GERD=Gastro-Esophageal Reflux Disease, OSA= Obstructive Sleep Apnea, PAD= peripheral artery disease, PH= Pulmonary Hypertension.*

*Substance use disorder* comprise alcohol, hallucinogens, stimulants, sedatives and/or opioids use

e-Figure 2. Kaplan-Meier survival curve comparing the three groups. In blue is the control group, red for “Young COPD” and green for the older COPD.


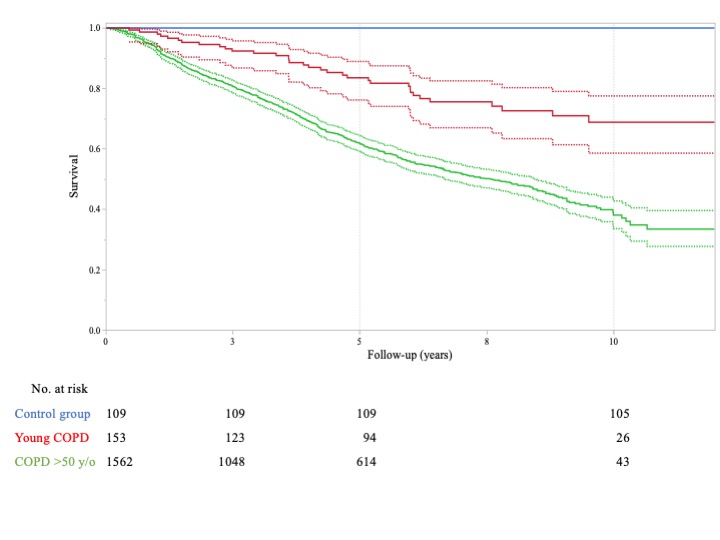


The solid line represents the survival curve, dotted lines represent the 95% confidence interval.

Vital status is missing in 16 controls, 7 “Young COPD”, and 294 older COPD.

The six recorded death in the control group occurred after 16 years of observation.

e-Figure 3. Primary causes of death in the Young (*<* 50 years) and “Older” COPD patients (>50 years).


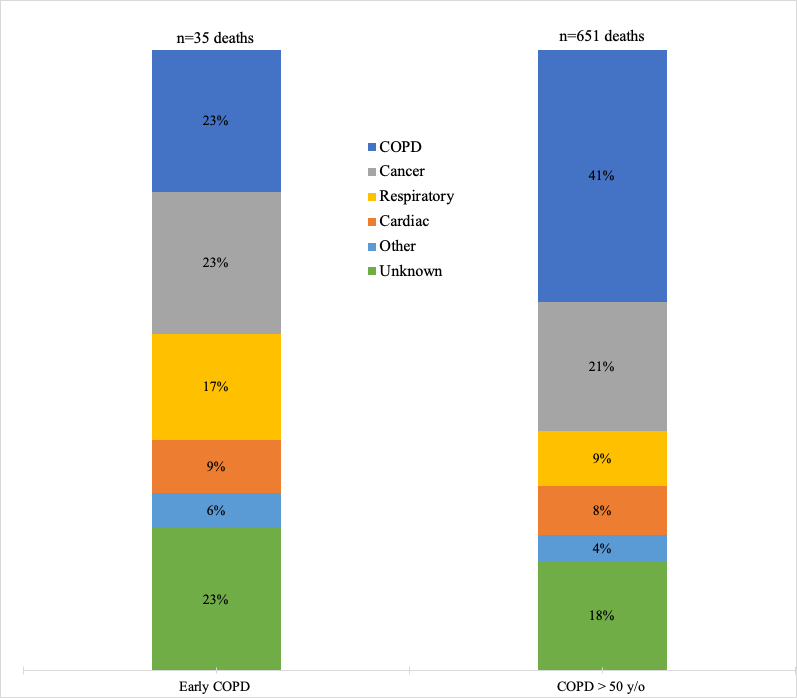


Young COPD

E-Table 1. List of comorbidities with at least 1% prevalence, comparing Young COPD with Controls.

| **Comorbidity** | **Young COPD** | **Controls** | **Prob>ChiSq** |
| --- | --- | --- | --- |
| **Hyperlipidemia*** | 25% | 10% | **0.0457** |
| **Hypertension*** | 24% | 7% | **0.0259** |
| **DJD*** | 18% | 2% | **0.0302** |
| **Substance use disorder*** | 17% | 0% | **0.0005** |
| **Depression*** | 15% | 5% | **0.0325** |
| GERD | 14% | 3% | 0.0653 |
| CAD | 13% | 2% | 0.307 |
| Anxiety | 9% | 6% | 0.4784 |
| Diabetes Mellitus | 9% | 5% | 0.2015 |
| **Hepatitis*** | 9% | 0% | **0.0046** |
| BPH | 7% | 2% | 0.3097 |
| **Bipolar disorder*** | 6% | 0% | **0.0187** |
| **CRF*** | 6% | 0% | **0.0013** |
| OSA | 6% | 3% | 0.2682 |
| Gastro-Duodenal ulcers | 5% | 1% | 0.5332 |
| Lung cancer | 4% | 0% | 0.219 |
| **Tuberculosis*** | 4% | 1% | **0.0439** |
| Erectile dysfunction | 3% | 0% | 0.1597 |
| Prostate cancer | 3% | 2% | 0.4314 |
| Nephrolithiasis | 3% | 2% | 0.6443 |
| **Osteoporosis*** | 3% | 0% | **0.0336** |
| Venous insufficiency | 3% | 2% | 0.3536 |
| Cervical cancer | 3% | 0% | 0.1519 |
| *Asthma****§*** | 3% | 7% | 0.0764 |
| Bronchiectasis | 3% | 1% | 0.2291 |
| Cataract | 3% | 0% | 0.2688 |
| CVA | 3% | 0% | 0.2228 |
| DVT | 3% | 0% | 0.1567 |
| **Gout*** | 3% | 0% | **0.0417** |
| Hypothyroidism | 3% | 1% | 0.2291 |
| Liver cirrhosis | 3% | 1% | 0.2291 |
| PAD | 3% | 1% | 0.2291 |
| Gallbladder disease | 2% | 1% | 0.3945 |
| Pulmonary Hypertension | 2% | 0% | 0.0556 |
| Pulmonary fibrosis | 2% | 1% | 0.3945 |
| Mitral Valvulopathy | 2% | 0% | 0.0775 |
| CHF | 1% | 0% | 0.1185 |
| CTD | 1% | 5% | 0.0847 |
| DM with Neuropathy | 1% | 0% | 0.1185 |
| Glaucoma | 1% | 0% | 0.1185 |
| Pancreatitis | 1% | 1% | 0.6677 |
| RLS | 1% | 0% | 0.1185 |
| Seizure | 1% | 0% | 0.1185 |
| Ventricular arrhythmias | 1% | 0% | 0.1185 |
| Atrial Fibrillation | 1% | 1% | 0.8971 |
| Bladder cancer | 1% | 1% | 0.8971 |
| Bronchiolitis | 1% | 0% | 0.2703 |
| Dementia | 1% | 0% | 0.2703 |
| Head & Neck cancer | 1% | 1% | 0.8971 |
| HIV | 1% | 0% | 0.2703 |
| Kidney cancer | 1% | 0% | 0.2703 |
| Macular degeneration | 1% | 0% | 0.2703 |
| MAI | 1% | 0% | 0.2703 |
| Pancreatic cancer | 1% | 0% | 0.2703 |
| Pulmonary embolism | 1% | 0% | 0.2703 |
| Celiac disease | 0% | 1% | 0.2086 |
| Colon cancer | 0% | 1% | 0.2086 |
| Thyroid Cancer | 0% | 2% | 0.0749 |

The asterisk (*) denote those diseases with a statistically significant difference based on the logistic regression, testing the hypothesis that differences in prevalence are due group membership when adjusting for age, gender and cumulative smoking.

***§*** *In the COPD groups, asthma diagnosis was accounted for those participants recruited after 2010, as it was an exclusion criterion in the initial BODE cohort protocol.*

*Abbreviations: AAA= Abdominal Aortic Aneurism, BPH= Benign Prostatic Hypertrophy, CA= Cancer, CAD = Coronary Artery Disease, CHF=Congestive Heart Failure, CRF=Chronic Renal Failure, CTD= Connective Tissue Disorders, CVA=Cerebro-Vascular Accident, DJD= Degenerative joint Disease, DVT= Deep Venous Thrombosis, GERD=Gastro-Esophageal Reflux Disease, MAI= Mycobacterium Avium-Intracellulare, OSA= Obstructive Sleep Apnea, PAD= peripheral artery disease, PH= Pulmonary Hypertension, RLD= Restless Leg Syndrome.*

E-Table 2. List of comorbidities with at least 1% prevalence, comparing Young with Old COPD patients.

| **Comorbidity** | **Old COPD**  **(>50 years)** | **Young COPD**  **(*≤* 50 years)** | ***p-value*** |
| --- | --- | --- | --- |
| ***Hypertension**** | 53% | 24% | <0.0001 |
| **Hyperlipidemia*** | 43% | 25% | <0.0001 |
| **BPH*** | 30% | 7% | <0.0001 |
| **CAD*** | 27% | 13% | 0.0007 |
| DJD | 27% | 18% | 0.1103 |
| **Diabetes Mellitus*** | 21% | 9% | 0.0043 |
| **PAD*** | 16% | 3% | <0.0001 |
| **CHF*** | 15% | 1% | 0.0011 |
| **CRF*** | 15% | 6% | 0.0032 |
| Depression | 15% | 15% | 0.9888 |
| GERD | 15% | 14% | 0.6995 |
| **Atrial Fibrillation*** | 13% | 1% | <0.0001 |
| Anxiety | 12% | 9% | 0.2742 |
| **CVA*** | 11% | 3% | 0.0019 |
| Gastro-Duodenal ulcers | 11% | 5% | 0.0815 |
| Substance use disorder | 11% | 17% | 0.3185 |
| **Erectile dysfunction*** | 10% | 3% | 0.0008 |
| Cataract | 9% | 3% | 0.0590 |
| OSA | 9% | 6% | 0.3487 |
| **Pulmonary hypertension*** | 9% | 2% | 0.0342 |
| Lung cancer | 8% | 4% | 0.2246 |
| **Osteoporosis*** | 8% | 3% | 0.0333 |
| Prostate cancer | 8% | 3% | 0.3525 |
| Bipolar disorder | 7% | 6% | 0.6706 |
| Breast cancer | 7% | 0% | 0.2865 |
| **Hypothyroidism*** | 7% | 3% | 0.0480 |
| **AAA*** | 6% | 0% | <0.0001 |
| DVT13 | 5% | 3% | 0.2107 |
| Pulmonary fibrosis | 5% | 2% | 0.4513 |
| Ventricular arrhythmias | 5% | 1% | 0.7761 |
| Gallbladder disease | 4% | 2% | 0.1276 |
| **Glaucoma*** | 4% | 1% | 0.0186 |
| Gout | 4% | 3% | 0.5132 |
| Tuberculosis | 4% | 4% | 0.9112 |
| Venous insufficiency | 4% | 3% | 0.7123 |
| **Aortic Valvulopathy*** | 3% | 0% | 0.0044 |
| Bladder cancer | 3% | 1% | 0.0524 |
| DM w/ Neuropathy | 3% | 1% | 0.1252 |
| **Hepatitis§** | 3% | 9% | 0.0015 |
| Nephrolithiasis | 3% | 3% | 0.8563 |
| **Asbestosis*** | 2% | 0% | 0.0258 |
| Asthma | 2% | 3% | 0.4301 |
| Bronchiectasis | 2% | 3% | 0.5236 |
| **Colon cancer*** | 2% | 0% | 0.0245 |
| CTD | 2% | 1% | 0.381 |
| Dementia | 2% | 1% | 0.125 |
| Head & Neck cancer | 2% | 1% | 0.2365 |
| Liver cirrhosis | 2% | 3% | 0.6538 |
| Mitral V | 2% | 2% | 0.7071 |
| Pancreatitis | 2% | 1% | 0.7548 |
| Pulmonary embolism | 2% | 1% | 0.2055 |
| Seizure | 2% | 1% | 0.4994 |
| Alzheimer | 1% | 0% | 0.1908 |
| Hyperthyroidism | 1% | 0% | 0.1264 |
| Hypogonadism | 1% | 0% | 0.1414 |
| IBD | 1% | 0% | 0.1399 |
| Kidney cancer | 1% | 1% | 0.8417 |
| Leukemia | 1% | 0% | 0.1399 |
| Macular degeneration | 1% | 1% | 0.5746 |
| MAI | 1% | 1% | 0.9047 |
| Melanoma | 1% | 0% | 0.0774 |
| Parkinson | 1% | 0% | 0.1264 |
| RLS | 1% | 1% | 0.6786 |
| Sarcoidosis | 1% | 0% | 0.1718 |
| Broncholitis | 0% | 1% | 0.226 |
| Cervical cancer | 0% | 3% | 0.0506 |
| HIV | 0% | 1% | 0.5595 |
| Pancreatic cancer | 0% | 1% | 0.5369 |

The asterisk (*) denote those diseases where the prevalence is significantly higher in the older COPD. The (**§)** denote those diseases where the prevalence is significantly higher in the Young COPD.

*Abbreviations: AAA= Abdominal Aortic Aneurism, BPH= Benign Prostatic Hypertrophy, CA= Cancer, CAD = Coronary Artery Disease, CHF=Congestive Heart Failure, CRF=Chronic Renal Failure, CTD= Connective Tissue Disorders, CVA=Cerebro-Vascular Accident, DJD= Degenerative joint Disease, DVT= Deep Venous Thrombosis, GERD=Gastro-Esophageal Reflux Disease, IBD=Inflammatory Bowel Disease, MAI= Mycobacterium Avium-Intracellulare, OSA= Obstructive Sleep Apnea, PAD= peripheral artery disease, PH= Pulmonary Hypertension, RLD= Restless Leg Syndrome.*
